# Supplementary material for: EpidermaQuant: Unsupervised Detection and Quantification of Epidermal Differentiation Markers on H-DAB-Stained Images of Reconstructed Human Epidermis
Source: Diagnostics (Basel). 2024 Aug 29;14(17):1904. doi: 10.3390/diagnostics14171904 (PMC11394256; doi:10.3390/diagnostics14171904)
Supplement: Supplementary file 1 [file diagnostics-14-01904-s001.zip › diagnostics-3132369-supplementary.pdf]

Supplementary text

EpidermaQuant framework

The culmination of the work on the algorithm was the implementation of an application with a user-friendly graphical interface (see **Figure S1**). The User may preview the submitted image, the outcome and the percentage of DAB-stained tissue using the EpidermaQuant, also lets them choose the right image based on the marker applied in the study. The application has been written using the MATLAB® R2021b programming environment with a possibility of further development.

Color normalization and deconvolution

Vector matrix for *ImageJ Color Deconvolution 2 plug* in implementation:

MODx = [0.650, 0.268, 0]

MODy = [0.704, 0.570, 0]

MODz = [0.286, 0.776, 0]

Different variants of the matrix were tested for color deconvolution using the *Python scikit-image* implementation method:

- matrix 1:

He = [0.6500286; 0.704031; 0.2860126]

DAB = [0.26814753; 0.57031375; 0.77642715]

Res = [0.7110272; 0.42318153; 0.5615672]

- matrix 2:

He = [0.18; 0.20; 0.08]

DAB = [0.10; 0.21; 0.29]

Res = [0.01; 0.13; 0.01]

- matrix 3:

He = [0.651; 0.701; 0.290]

DAB = [0.269; 0.568; 0.778]

Res = [0.633; -0.713; 0.302]

Supplementary figures

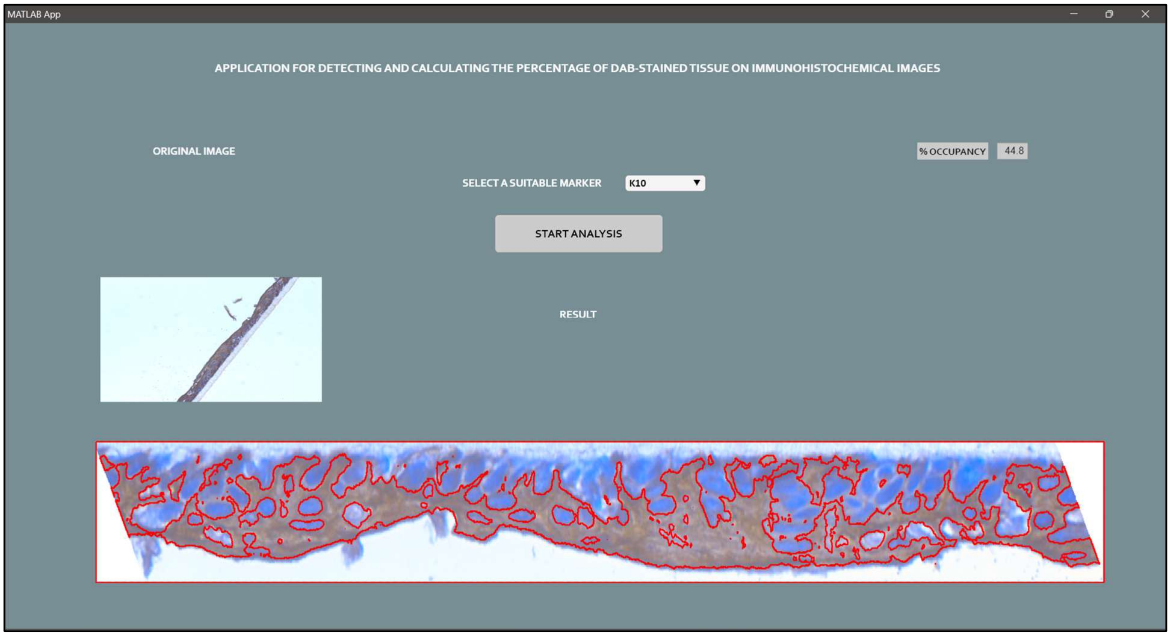

Figure S1: EpidermaQuant application. *The graphical interface allows user to select the appropriate image by the marker used in the analysis and to preview the image entered, the final result and the percentage of DAB-stained tissue.*

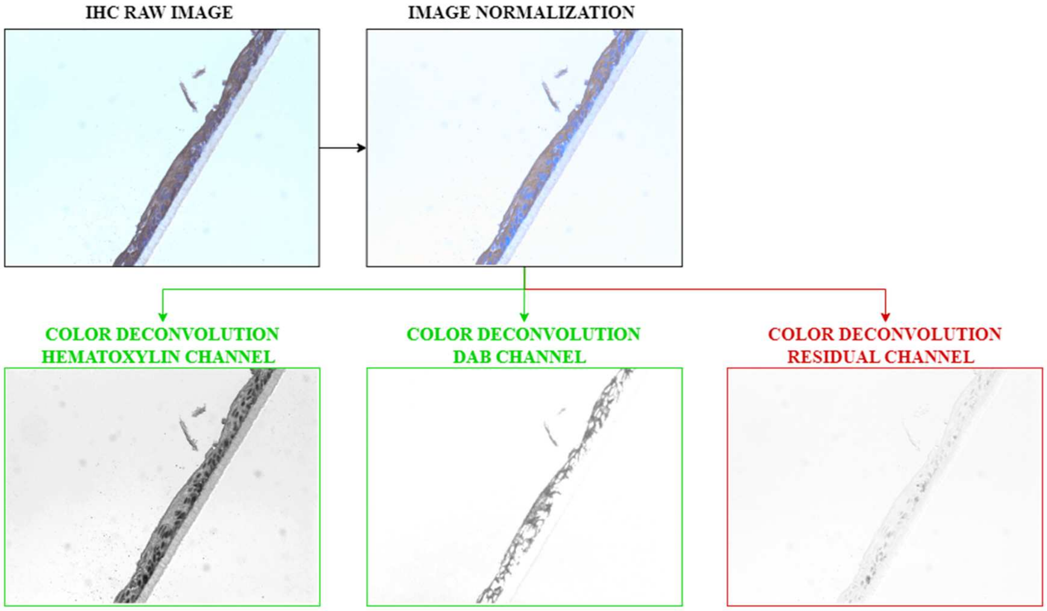

Figure S2: Color normalization and deconvolution of IHC image. *The first step of image processing is color normalization which reduces the variability of pixel intensity values in different samples. In the color deconvolution step, the RGB image is split into three channels, corresponding to the colors of the stains used (the residual-channel image is not further processed).*

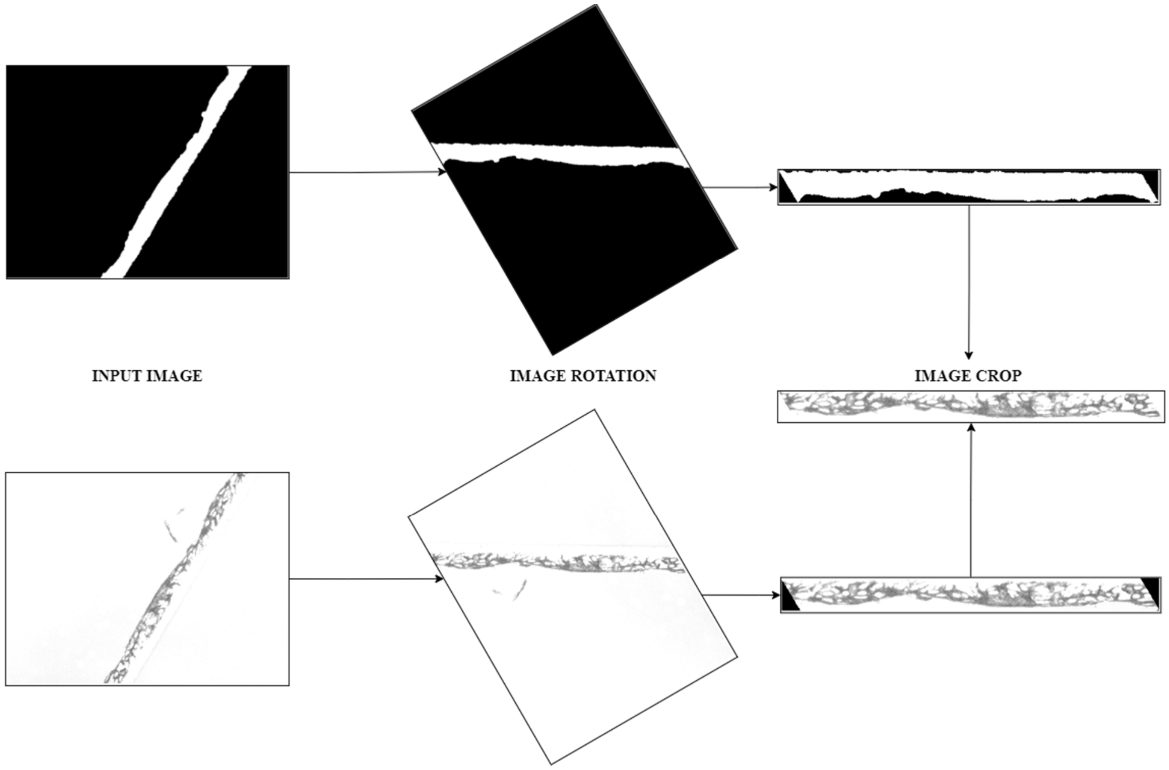

__Figure S3: Automatic image rotation and crop.__ Based on the rotated and cropped mask, it is possible to scale the DAB-channel image accordingly. It allows us to remove the unnecessary in further analysis background. Rotation is performed by its method based on the boundary distribution of image intensity.

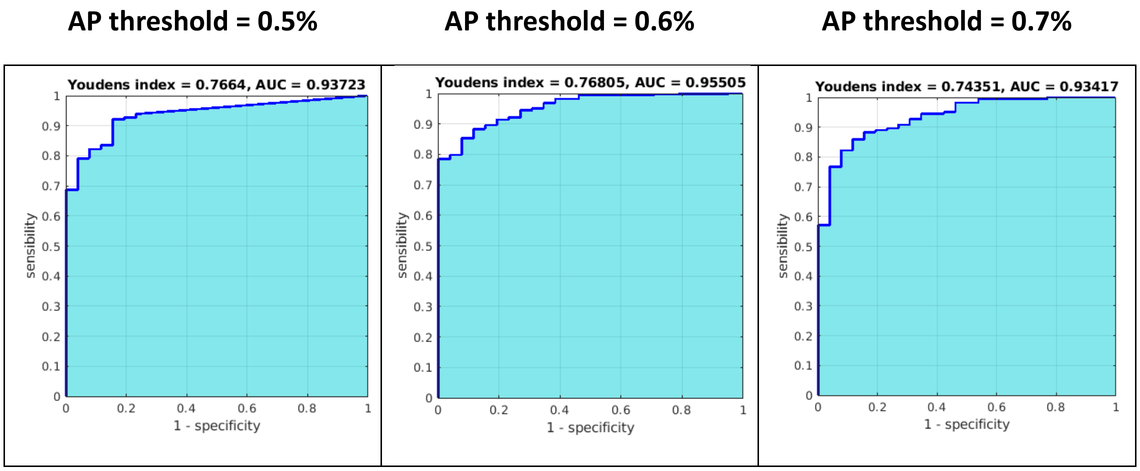

__Figure S4: Summary of determining the optimal threshold value results.__ Comparison of potential threshold values based on ROC curves, including results of AUC and Youden index.

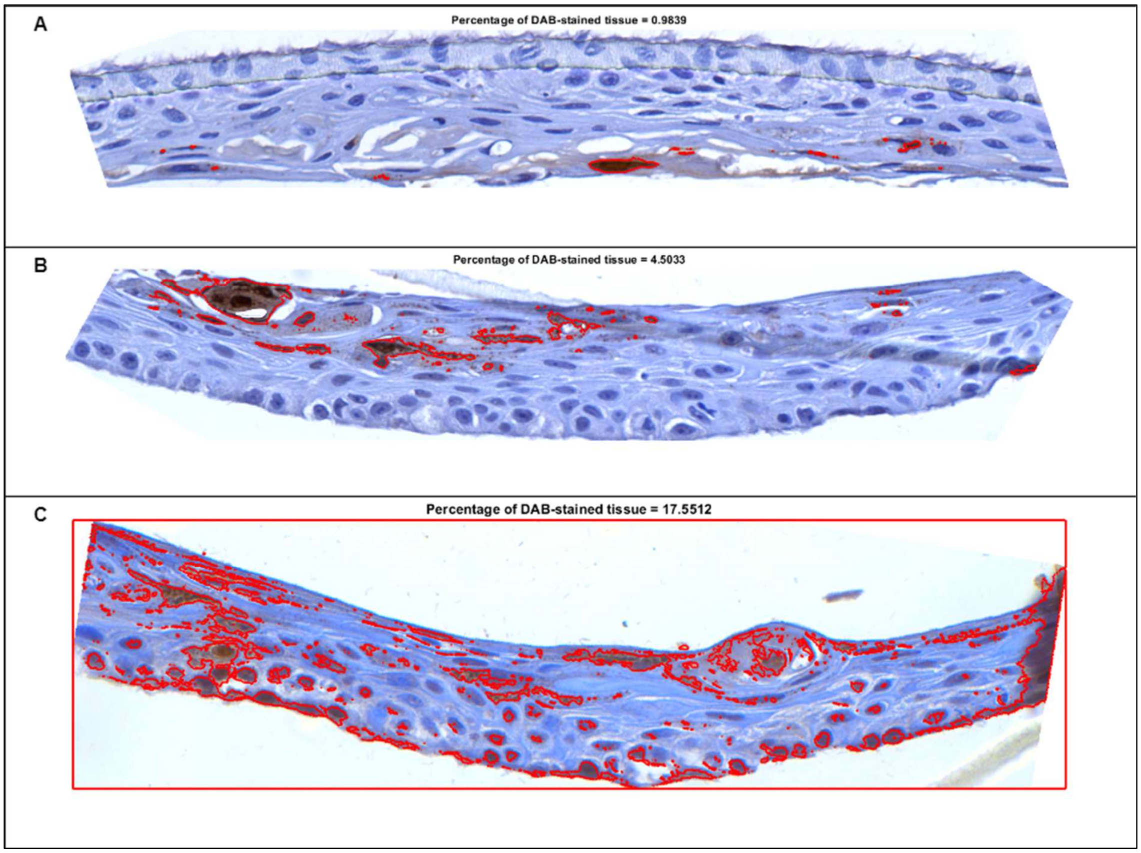

Figure S5: Final output of the algorithm for example images representing the FLG marker. *Percentage of DAB occupancy on the tissue with the outlines of the DAB areas marked over the original image.*

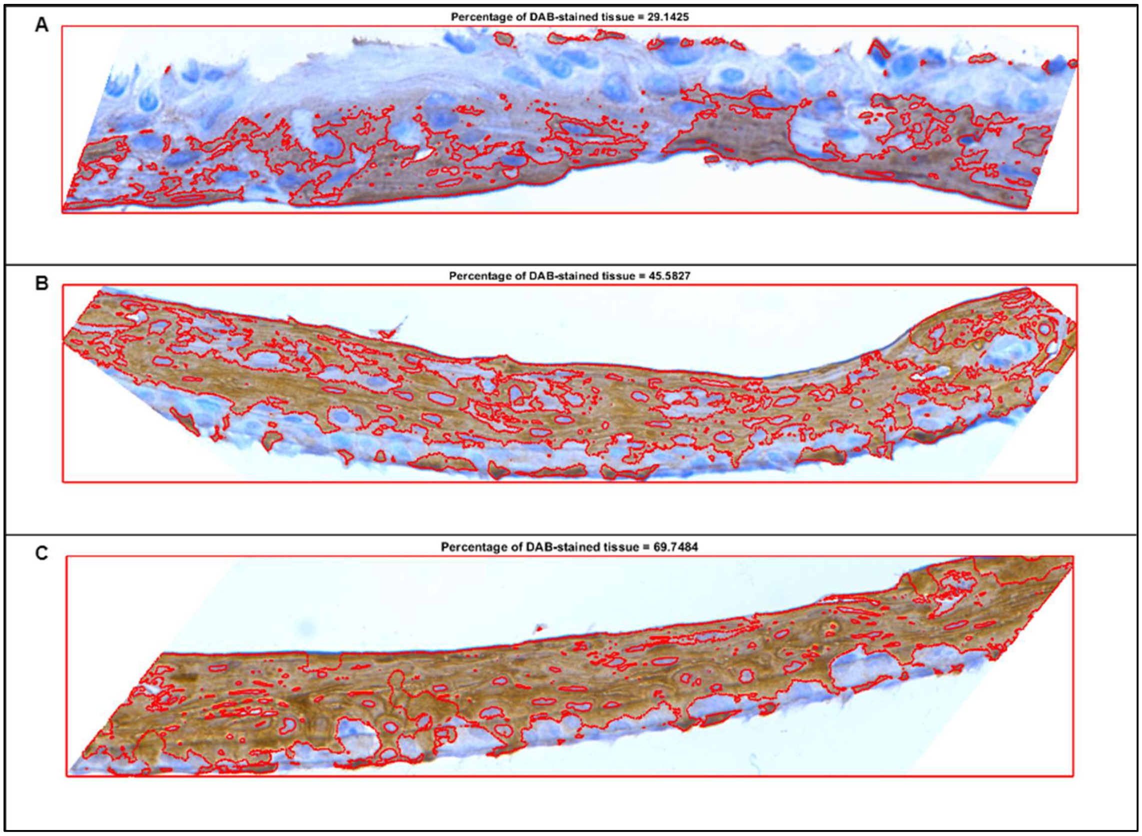

Figure S6: Final output of the algorithm for example images representing the K10 marker. *Percentage of DAB occupancy on the tissue with the outlines of the DAB areas marked over the original image.*

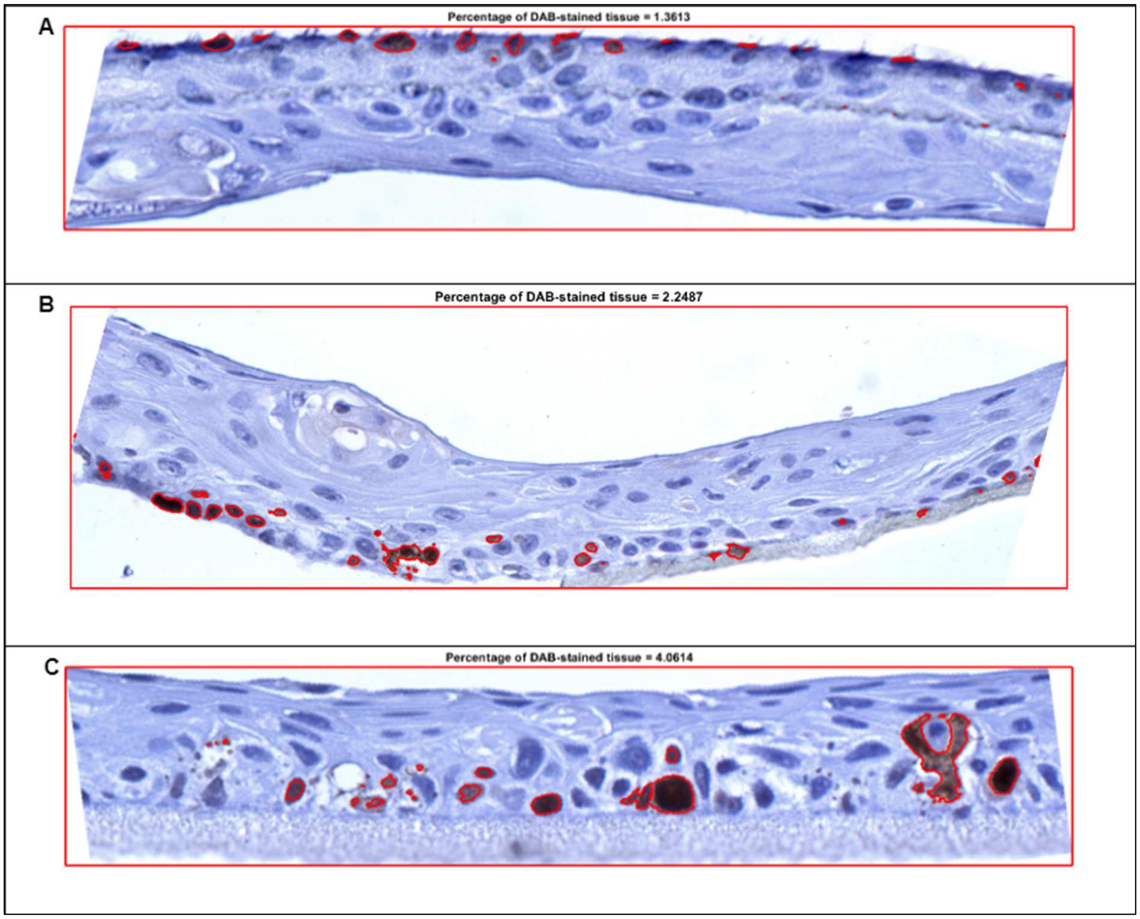

Figure S7: Final output of the algorithm for example images representing the Ki67 marker. *Percentage of DAB occupancy on the tissue with the outlines of the DAB areas marked over the original image.*

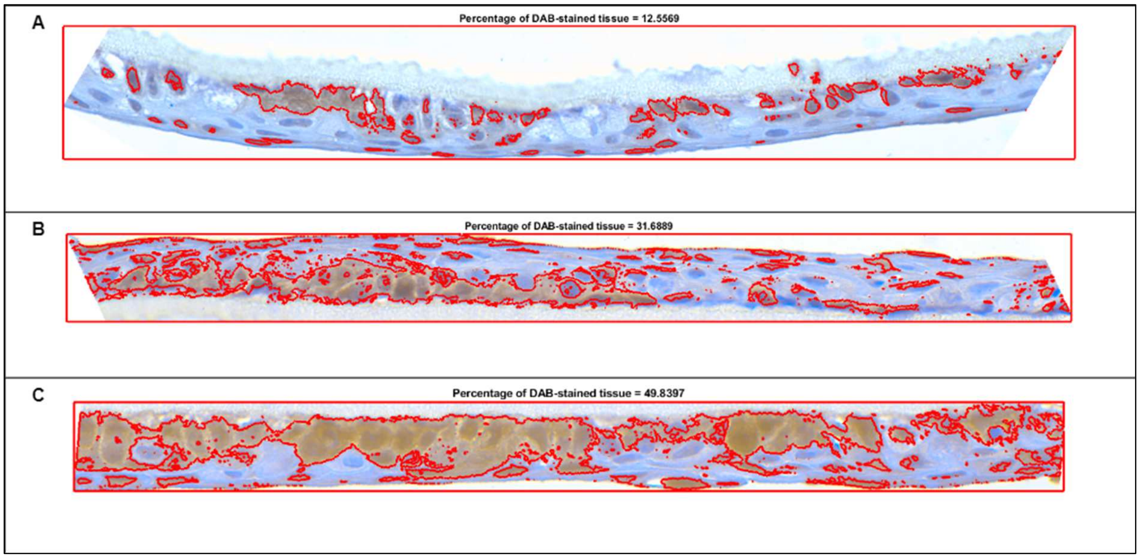

Figure S8: Final output of the algorithm for example images representing the HSPA2 marker. *Percentage of DAB occupancy on the tissue with the outlines of the DAB areas marked over the original image.*

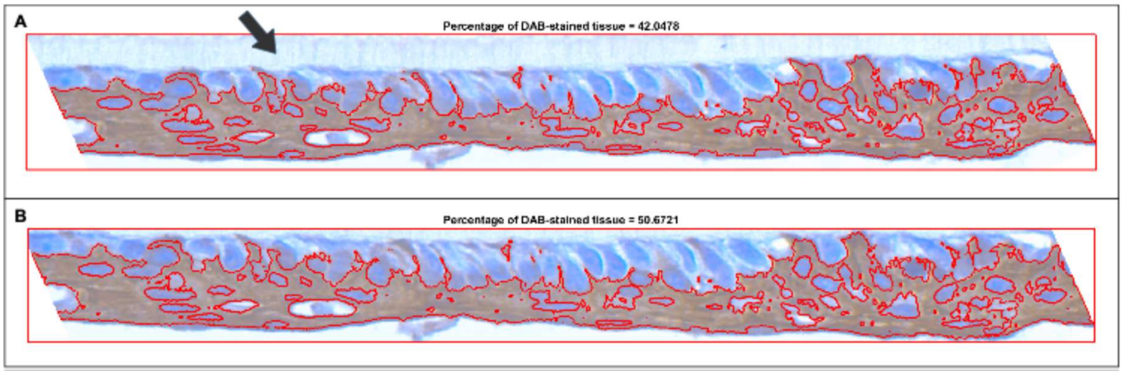

Figure S9: Final output of the algorithm for example image with a plastic membrane (indicated by an arrow symbol) representing the K10 marker. *Percentage of DAB occupancy on the tissue with the outlines of the DAB areas marked over the original image before (A) and after (B) removing the plastic culture medium.*

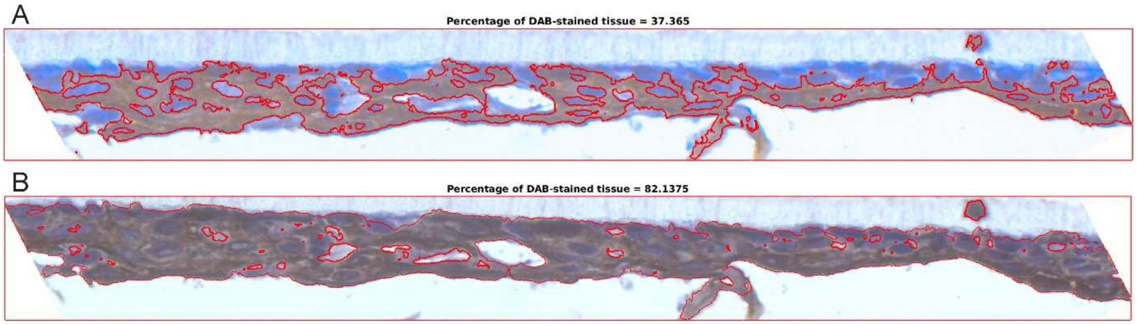

Figure S10: Final output of the algorithm for example image with and without image normalization representing the K10 marker. *Percentage of DAB occupancy on the tissue with the outlines of the DAB areas marked over the original image with (A) and without (B) normalization.*
